# Exposure to the field of renal transplantation during undergraduate medical education in the UK

**DOI:** 10.1186/1472-6920-5-32

**Published:** 2005-09-14

**Authors:** Anusha G Edwards, Alex Newman, Justin D Morgan

**Affiliations:** 1 Department of Surgery, Southmead Hospital, Westbury on Trym, Bristol BS10 5NB, UK

## Abstract

**Background:**

There is a lack of surgeons in the field of renal transplantation, with a predicted shortage of over 20 consultants by the year 2005. Early positive exposure to the field, commencing at undergraduate level, has been identified as being vital to improving rates of recruitment. This study was performed to assess the exposure of undergraduates to the field of renal transplantation during medical education in the UK.

**Methods:**

In October 2004 a questionnaire was sent to the clinical deans of all UK medical schools regarding undergraduate exposure to renal transplantation.

**Results:**

Twenty-five replies were received, giving a response rate of 96%. All but one school had a centre for renal transplantation in their region. Three schools (12%) gave no formal lecture or tutorial on the subject during the entire course. Of the remainder, between one to four formal sessions were provided, ranging from 15 minutes to 3 hours duration.

Six medical schools (24%) provided no compulsory clinical exposure to renal transplantation, with a further five (20%) saying that students may receive exposure by chance. The average length of attachment was three weeks. Twenty-one medical schools (84%) provided between 1–10% of students a choice to study renal transplantation, as part of electives and special study modules.

**Conclusion:**

This study reveals a variation between, and within, medical schools in the levels of formal teaching. If the trends in recruitment to renal transplantation are to be reversed, we have an obligation to improve upon the medical education that students currently receive.

## Background

Renal transplantation is the first choice treatment for many patients with end-stage renal failure, with the potential for improved quality of life and increased life expectancy [1,2]. However, in the UK, the speciality suffers from a lack of qualified surgeons with a predicted shortage, in 1999, of over twenty consultant renal transplant surgeons by the year 2005 [3]. The most recent figures have shown that of the 94 renal transplant consultant posts in the UK, 12 are filled by locums [4].

Previous work has highlighted the multiple factors that deter surgical trainees from the speciality, which include a lack of exposure to the speciality, at an early stage during training [3]. This has lead to a call for the inclusion of transplantation within basic surgical training rotation programmes.

Last year a crisis meeting, organised by the British Transplantation Society, (BTS) was held to address the problem with recruitment into renal transplantation. It was identified that early, positive exposure to the field should be commenced at undergraduate level and a number of ways of addressing this were suggested [5]. A recent study, at a single UK medical school demonstrated a lack of exposure to and knowledge of renal transplantation [6], but no literature exists regarding the national situation. Therefore, this survey was conducted to assess how much exposure to the field of transplantation medical students in the UK are currently receiving.

## Methods

**Figure 1:**
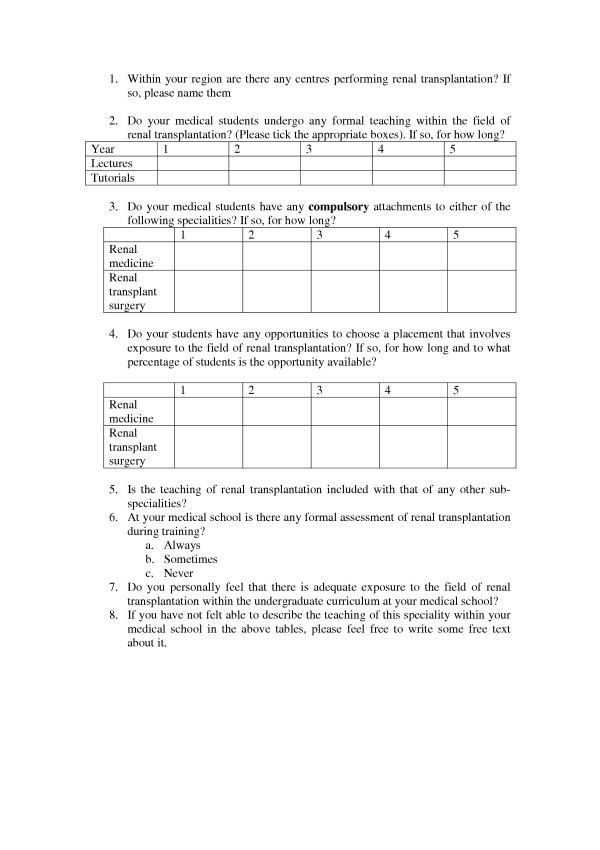
Questionnaire distributed to clinical deans of all UK medical schools.

In October 2004 a questionnaire was sent to the clinical deans of all 26 UK medical schools. It consisted of eight questions regarding exposure of undergraduates to the field of renal transplantation. (Figure 1) Although not validated, it was based upon a questionnaire from a similar study in the field of ENT [7]. Non-responders were sent a further copy of the questionnaire after four weeks, which were followed with a telephone call after a further four weeks, if necessary.

## Results

Replies were received from twenty-five medical schools, giving a response rate of 96%. However, one response was incomplete as the clinical dean felt that the course was "too integrated" to be analysed in detail.

**Figure 2:**
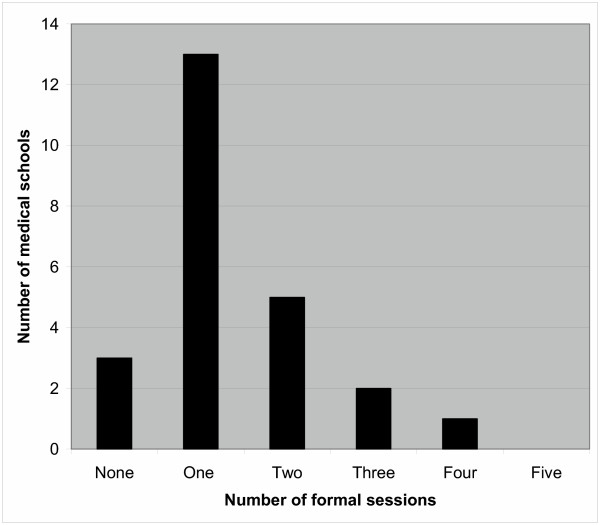
A plot of the number of formal teaching sessions provided by UK medical schools.

Three schools (12%) gave no formal lecture or tutorial on the subject during the entire undergraduate course. Of the remainder, between one to four formal sessions were provided, ranging from 15 minutes to 3 hours duration (Figure 2). Two of these medical schools specified that formal sessions were only available to students attached to a transplantation firm, but gave no indication of what proportion of students this applied to.

Despite the fact that all but one of the responding medical schools had a centre for renal transplantation in their region six medical schools (24%) provided no compulsory clinical exposure to renal transplantation, with a further five (20%) saying that students may receive exposure by chance. The average length of attachment was three weeks, ranging from one to five weeks. However, twenty-one medical schools (84%) provided between 1–10% of students a choice to study renal transplantation, usually as part of electives and special study modules. The duration of such options ranged from two to four weeks.

The majority of medical schools, fifteen, (60%), included the teaching of renal transplantation with other specialities, whilst one school felt that the course was too integrated to tell. (table 1)

**Table 1:** The number of medical schools teaching renal transplantation in conjunction with a range of other subjects

**Subject**	**Number of medical schools**
Renal medicine	8
Surgery	3
Urology	2
Pathology	1
Immunology	4
Transplant medicine	1
Ethics	2

Regarding the assessment of knowledge of renal transplantation, one medical school always (4%), twelve schools sometimes (48%) and nine never (36%) formally assesses it during examinations.

Fifteen of the responding clinical deans (60%) felt that there was adequate exposure to renal transplantation in the undergraduate curriculum. Of the remainder, one thought there wasn't, two were unsure, three found it difficult to find teachers and three thought that it is, and should remain a postgraduate subject.

## Discussion

This study reveals a worrying variation both between, and within, medical schools in the levels of formal teaching and exposure to renal transplantation. There is no data available regarding the correlation between level of exposure to transplantation and final career choice. However, previous work has demonstrated that one factor that deters surgical trainees from a career in transplantation is a lack of exposure to the speciality [3]. We therefore believe that if the trends in recruitment to the field are to be reversed, everyone working in the speciality has an obligation to improve upon the medical education that students currently receive in the UK.

This work is the first formal assessment of exposure to renal transplantation in UK medical schools. The excellent response rate makes this a comprehensive study. However, the fact that one response was incomplete suggests that with integrated courses assessing the exposure to one speciality can be difficult. Although the questionnaire was not formally validated, it was designed to be used in today's medical schools, and was based upon a questionnaire used in a similar study assessing undergraduate exposure to ENT surgery [8].

A previous study performed at a single medical school, with two local centres of renal transplantation, demonstrated variability in the amount of exposure to transplantation and worryingly low levels of knowledge about the field, amongst final year medical students [6]. This national study suggests that similar or lower levels of exposure are occurring throughout the country, which has worrying implications for the future of renal transplantation, both in terms of recruitment and organ procurement.

It has been shown that increased knowledge about organ donation is associated with an increased likelihood of holding an organ donor card and feeling more comfortable in approaching relatives of potential organ donors [9]. In the UK the speciality also suffers from an ever-increasing discrepancy between the number of organs donated and the number of patients on the transplant waiting list [10]. Over the last few years non heart-beating programmes have been introduced in the UK, in order to increase the number of organs available. They can potentially increase the transplant rate by 20–40% [11]. However, if the doctors of tomorrow are not aware of such programmes and do not feel equipped to approach relatives, levels of donation are unlikely to be maximised.

Obviously, there are other factors that deter trainees from a career in transplantation [3] and these too must be tackled in order to reverse the current recruitment trends. These include the significant out-of-hours commitment and appropriate recompense, both of which are currently being tackled within the frameworks of the European Working Time Directive and the new consultant contracts respectively. Career progression and training are also issues of concern for trainees, which have been partly dealt with by the recent provision of funding by the Department of Health for more specialist registrar training posts in renal transplantation [4].

One clinical dean suggested that the F2 year might be an opportunity to give students a "taster" in the speciality. This proposal has also been considered by the BTS, with an F2 year, consisting of 4 months each of renal transplantation, nephrology and general surgery or urology. The BTS is currently liasing with the national Modernising Medical Careers group about this option and individual transplant units have been encouraged to submit appropriate bids to their Postgraduate Dean.

The recommendations made by the BTS crisis meeting for early positive exposure at the undergraduate level included a presence at medical school careers fairs and surgical societies, along with the introduction of a full time educator at UK Transplant [12]. Such measures should help to raise the profile of the speciality. Indeed studies in the USA have demonstrated that positive encounters with surgeons can favourably influence the perceptions of first year medical students towards a career in surgery [13]. However, an early interest must be fostered throughout undergraduate training if students are to seriously consider transplantation as a career. For many doctors this comes from actually witnessing the speciality at work, receiving good, practical teaching [14] or being inspired by an individual in the field [15]. Therefore, the authors believe that as teaching time is limited during undergraduate studies medical schools should ensure that the person chosen to teach renal transplantation is someone who is able to communicate well with students and can present the speciality in such a way as to inspire. This individual does not necessarily have to be a transplant surgeon as the subject can also be taught in conjunction with other subjects such as renal medicine and immunology. In addition to conventional bedside teaching and lectures the multi disciplinary nature of transplantation should be exploited. Involving students in patient education days and the writing of information sheets or patient web pages may prove more memorable and inspirational to some students than conventional forms of exposure.

## Conclusion

This study has demonstrated that amongst medical schools in the UK there is considerable variation in the levels of exposure to the field of renal transplantation. Given the current shortage of renal transplant surgeons this is of great concern. In order to reverse the recruitment crisis all professionals involved in the speciality should take it upon themselves to increase exposure to and profile of the field.

## List of abbreviations

**UK **– United Kingdom

**BTS **– British Transplantation Society

**ENT **– Ear, nose and throat

**USA **– United States of America

## Competing interests

The author(s) declare that they have no competing interests.

## Authors' contributions

AE was involved in study design and execution and manuscript preparation.

AN was involved in questionnaire distribution and collection and manuscript preparation.

JM was involved in study design and manuscript preparation.

## Pre-publication history

The pre-publication history for this paper can be accessed here:


